# Does haste make waste? Prevalence and types of errors reported after publication of studies of COVID-19 therapeutics

**DOI:** 10.1186/s13643-023-02381-4

**Published:** 2023-11-16

**Authors:** Brittany Chatterton, Simon B. Ascher, Naihua Duan, Richard L. Kravitz

**Affiliations:** 1 Department of Internal Medicine, University of California, Davis, Sacramento, CA USA; 2 Center for Healthcare Policy and Research, University of California, Davis, Sacramento, CA USA; 3 Division of Mental Health Data Science, Department of Psychiatry, Columbia University, New York City, NY USA

**Keywords:** Errata, Erratum, COVID-19 therapeutics, COVID-19 treatment

## Abstract

**Background:**

The COVID-19 pandemic spurred publication of a rapid proliferation of studies on potential therapeutic agents. While important for the advancement of clinical care, pressure to collect, analyze, and report data in an expedited manner could potentially increase the rate of important errors, some of which would be captured in published errata. We hypothesized that COVID-19 therapeutic studies published in the early years of the pandemic would be associated with a high rate of published errata and that, within these errata, there would be a high prevalence of serious errors.

**Methods:**

We performed a review of published errata associated with empirical studies of COVID-19 treatments. Errata were identified via a MEDLINE and Embase search spanning January 2020 through September 2022. Errors located within each published erratum were characterized by location within publication, error type, and error seriousness.

**Results:**

Of 47 studies on COVID-19 treatments with published errata, 18 met inclusion criteria. Median time from publication of the original article to publication of the associated erratum was 76 days (range, 12–511 days). A majority of errata addressed issues with author attribution or conflict of interest disclosures (39.5%) or numerical results (25.6%). Only one erratum contained a serious error: a typographical error which could have misled readers into believing that the treatment in question had serious adverse effects when in fact it did not.

**Conclusions:**

Despite accelerated publication times, we found among studies of COVID-19 treatments the majority of errata (17/18) reported minor errors that did not lead to misinterpretation of the study results. Retractions, an indicator of scientific misdirection even more concerning than errata, were beyond the scope of this review.

**Supplementary Information:**

The online version contains supplementary material available at 10.1186/s13643-023-02381-4.

## Introduction

Publication of biomedical research in scientific journals is the primary means of disseminating scientific findings to the research community and the public [1]. The goal is to accurately describe and interpret research findings, but mistakes are inevitable. Errors range from simple typographical glitches to more serious coding, arithmetic, or statistical errors that distort the study findings and lead to erroneous conclusions. Before publication, biomedical research reporting errors can be intercepted by authors, reviewers, or editors. Afterwards, the onus is on authors, journal editorial staff, and astute readers; noteworthy errors detected in this way are reported in the form of published errata, corrections, or corrigendum. While few studies have evaluated the incidence of such correctives, one report limited to general internal medicine and cardiology indicates they may be as frequent as 4.2 per 100 published articles [2].

In considering factors that may contribute to errors in the scientific record, one culprit may be pressure to communicate information rapidly, as might be seen when two competing research groups are rushing to claim a scientific discovery [3] or when external circumstances demand rapid reporting to support critical public health needs. The first 2 years of the COVID-19 pandemic created significant demand for research on therapies meant to inhibit the virus or mitigate the severity of clinical illness. Given the desperate need to understand the pathogenesis of COVID-19 and identify effective treatments, publications underwent rapid editorial review, with one international study reporting a nearly tenfold reduction in time from submission to acceptance for COVID-related compared with non-COVID related articles (11.3 versus 106 days) [4]. This accelerated schedule may have decreased the rigor of peer review.

Although several studies have evaluated retractions of COVID-19 publications [5–8], few have examined errors identified in published errata. We sought to characterize published errata related to COVID-19 treatment research and determine if compressed review times reported during the pandemic were associated with a higher prevalence and severity of error. We expected to find both a high rate of published errata and a high proportion of serious errors within published errata.

## Methods

We conducted a systematic review of errata published between January 1, 2020, and September 1, 2022, that were associated with articles reporting empirical studies of COVID-19 treatments. Errata published in English and related to COVID-19 therapeutics were identified on MEDLINE and Embase using the search terms COVID-19, erratum, corrigendum, and correction (the complete list of search terms, prepared with the assistance of a reference librarian, is provided in Additional file 1). Article-erratum pairs were included if the article’s topic was an empiric evaluation of COVID-19 treatment including head-to-head comparisons of treatment modalities, treatment versus placebo, or treatment versus standard of care or supportive care. Study types included clinical trials, observational studies, and systematic reviews. Articles were excluded if they investigated the treatment of another disease process during the COVID-19 pandemic, COVID-19 diagnostics, epidemiologic studies of the prevalence of treatment, or were editorial or opinion pieces on COVID-19 that did not present an empirical evaluation of a treatment.

Errata were categorized by location of the error in the original publication (abstract, body of the article, tables or figures, references, or supplemental material) and error type (author disclosure or conflict of interest, author attribution, numerical or statistical error, textual error, or citation error). Errors were then categorized as serious or minor. Errors were defined as serious if they (1) affected the study results in a way that could lead to erroneous conclusions by the author(s) or readers or (2) were found in the abstract or title, increasing the risk of error propagation. Two independent reviewers screened articles for inclusion and categorized errors identified in the errata. When discrepancies between reviewers arose (*n* = 3), the articles were jointly re-reviewed; all three were excluded by consensus. Data was managed in a REDCap [9] database and descriptive statistics were used to analyze data.

## Results

**Fig. 1:**
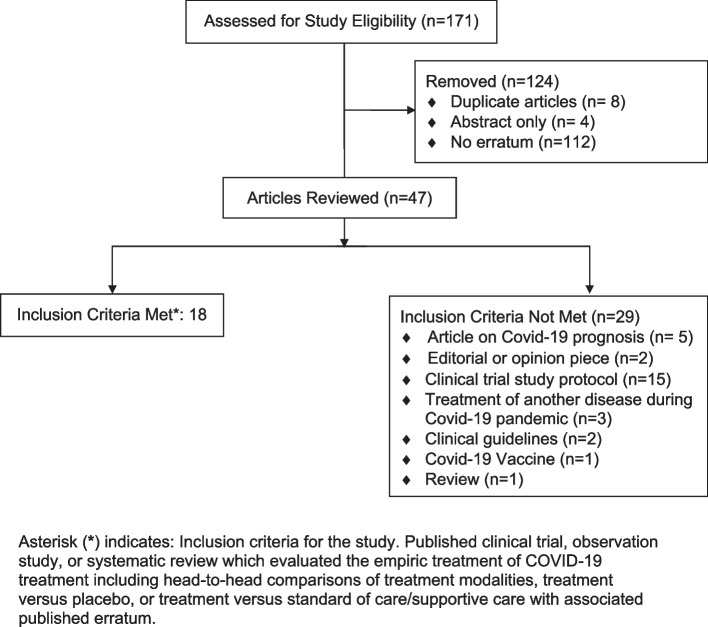
Flow diagram of article selection for study inclusion

The initial search produced 171 results; however, on review, 124 were removed because they (1) lacked an associated erratum (*n* = 112), (2) represented duplicate entries (*n* = 8), or (3) consisted of abstracts without full articles (*n* = 4) (Fig. 1). Of the remaining 47 article-erratum pairs, 18 articles (19 associated errata) met inclusion criteria (1 article had 2 separate published errata). Fifteen of the 18 studies were randomized clinical trials [10–24], two were systematic reviews [25, 26], and one was an observational study [27].

Among the 19 errata, 55% addressed 1 error within the published article, 28% 2 to 4 errors, and 17% ≥ 5 errors. Higher impact journals appeared to be somewhat over-represented (Table 1). Two thirds had first authors based outside of Europe or North America. Errors were most often found in the abstract, title, or byline and frequently involved author misattribution (16.3%) or failure to disclose potential conflicts of interest (23.3%) (Table 2). Median time from publication of the original article to publication of the associated erratum was 76 days (range 12 to 511 days). Among the errors identified, only one was characterized as serious (Table 3). This serious error was a typographical error in the Results section of the abstract. The sentence read, “There was severe adverse event recorded in the study group”; however, the correct statement was “There was no severe adverse event recorded in the study group.” This error would have led readers to question the safety of the study treatment, if the reader did not further investigate the body of the article which contained the correct results for the treatment group.

**Table 1:** Characteristics of published articles and errata on COVID-19 therapeutics research articles

		*N* (%)
**Articles, *N* = 18**	**Study type**	
	Clinical trial	15 (83.3)
	Systematic review	2 (11.1)
	Observational study	1 (5.5)
	**Year of publication**	
	2020	6 (33.3)
	2021	10 (55.5)
	2022	2 (11.1)
	**Journal impact factor**	
	0–2	4 (22.2)
	3–9	6 (33.3)
	> 10	8 (44.4)
	**Geographic region—first author**	
	Asia	9 (50)
	Europe	4 (22.2)
	North America	2 (11.1)
	South America	3 (16.6)
**Erratum, *N* = 19**	**Time to erratum publication (days)**	
	0–60	7 (36.8)
	61–120	6 (31.5)
	121–240	5 (26.3)
	> 240	1 (5.3)
	**Online publication corrected**	
	Yes	9 (47.3)
	No	10 (52.6)

**Table 2:** Characteristics of errors reported in published errata on COVID-19 therapeutics research articles

		**Error location**					
		Abstract/title/byline	Body of article	Tables and figures	Supplemental material	References	**Total *N* (%)**
**Error type**	Author attribution	4			3		**7 (16.3)**
	Author conflict of interest disclosure	10					**10 (23.2)**
	Numerical or statistical error	1	1	9			**11 (25.6)**
	Textual error		4				**4 (9.3)**
	Incorrect table or figure included		1				**1 (9.3)**
	Interpretation error^a^	1					**1 (9.3)**
	Citation error		2			1	**3 (7.1)**
	Other^b^	1		4	1		**6 (14.0)**
	**Total *N* (%)**	**17 (39.5)**	**8 (18.6)**	**13 (30.2)**	**4 (9.3)**	**1 (2.3)**	

^a^Analysis is correct, but the written explanation is incorrect

^b^Other category included the following: data file not included in publication, addition of new category of data (splitting treatment group into 2 groups), creation of new figures to visual data in original article, explanation of error as “minor error” without further explanation

**Table 3:** Sample of errors contained within errata for COVID-19 therapeutics articles

Error type	Minor vs serious	Prior to correction	After correction
Numerical/Statistical Error	Minor	Figure 1: 14 symptom onset > 3 days ago	Figure 1: 14 symptom onset > 7 days ago
Author attribution/disclosure	Minor	Affiliation for single author “Humanitas Research Hospital, Milan, Italy”	Correct affiliation: “Department of Biomedical Sciences, Humanitas University, Milan, Italy, and IRCCS Humanitas Research Hospital, Milan, Italy”
Interpretation error	Serious	“Results: There was severe adverse event recorded in the study group”	“Results: There was no severe adverse event recorded in the study group”
Textual error	Minor	“The subsection 3.1, titled '3.1. Data are mean (SD). Day-1 indicates baseline measurements' is a misprint and should be omitted”	“The statement 'Data are mean (SD). Day-1 indicates baseline measurements' corresponds to the legend of Fig. 2 of the original article”
Incorrect table/figure included	Minor	“Figures 3 and 4 were used twice due to a careless mistake during the preparation of Figures”	Correct Figures 3 and 4 now included in publication
Citation error	Minor	“As it has been proposed in an influenza model of antiviral candidate drugs evaluation”, should omit the “^25^” in superindex	“As it has been proposed in an influenza model of antiviral candidate drugs evaluation”
Other	Minor	Supplementary data file 1 containing anonymized patient data was inadvertently omitted	Supplementary data file 1 now included

## Discussion

In this study of published errata associated with articles on COVID-19 therapeutics, most reported errors were minor. Consistent with other studies, about half of detected errors were corrected in the original article’s online publication [2]. Many of these errors were related to author misattribution or failure to disclose conflict of interest. Others involved numerical errors that were primarily located in tables and figures. One serious error among 19 errata yields a serious error rate of 5%, which may lend cause for concern. This error, located in the abstract, misrepresented that the treatment group had serious adverse events, when in fact there were none. While we concede that therapeutic decisions are infrequently made on the basis of a single scientific study, a clinician quickly reviewing the abstract could have been dissuaded from pursuing what was found to be an effective COVID-19 therapeutic due to concerns of misreported adverse events with the treatment.

There have been several prior publications exploring reported error rates and seriousness of those errors. These studies have largely examined specific journals and included articles of all topics/types (Table 4). We observed a lower rate of serious errors (5%) than a study that reviewed randomized control trials from four high-impact journals (10%) [28]. When reviews expanded to include all study types, serious errors ranged from 14 to 25% of published errata [2, 29, 30]. One review of five radiology imaging journals found a very low overall errata rate (< 2%), with 6% of those errors found to be serious [31]. Another review dedicated to errors in authorship points out that each author is expected to proofread the manuscript prior to publication and thus errors related to name misspelling should be easily identified and corrected prior to publication [32]. That we found several errata related to misattribution of authorship—which ought to have been uncovered and corrected during the pre-publication process—raises the question as to whether other, less obvious errors were not identified. Additionally, this finding raises questions about the diligence of co-authors during the final pre-submission period.

**Table 4:** Summary of prior studies evaluating errata error rate in various journals and subject matters

Source	Year(s) examined	Journal(s) examined	Article type(s) included	Published errata rate	Errata categorized as serious
Bhatt, V et al. (2014) [33]	January 2012–December 2012	JAMA, Annals IM, BMJ, Lancet, NEJM	All article types	Mean 1.3 articles with ≥ 1 errata per issue	N/A
Hauptman, P et al. (2014) [2]	July 2009–December 2010	Top 10 general medicine and top 10 cardiology journals	Original studies, meta-analysis, reviews, guidelines, editorials/opinions, case reports, research letters	4.2 errata per 100 articles	24.4 per 100 errata
Castillo, M et al. (2011) [31]	June 2006–June 2011	JNM, Radiology, AJNR, AJR, RadioGraphics	All article types	1.77 errata per 100 articles	6.3 per 100 errata
Farrah, K and Rabb, D (2019) [29]	2015	Multiple journals	All studies included in 40 systematic reviews of drugs evaluated by the Canadian Agency for Drugs and Technologies in Health Common Drug Review	19 errata per 100 articles	16 per 100 errata
Molckovsky, A et al. (2011) [30]	2004–2007	JCO and JNCI	All article types	4 errata per 100 articles	14 per 100 errata
Royle, P and Waugh, N (2004) [28]	1995–2001	NEJM, JAMA, Lancet, BMJ	Randomized clinical trial	Lancet and JAMA: 8.4 per 100 articles NEJM: 8.3 per 100 articles BMJ: 5.6 per 100 articles	10 per 100 errata

*JAMA* Journal of the American Medical Association, *Annals IM* Annals of Internal Medicine, *BMJ* British Medical Journal, *NEJM* New England Journal of Medicine, *JNM* Journal of Nuclear Medicine, *AJNR* American Journal of Neuroradiology, *AJR* American Journal of Roentgenology, *JCO* Journal of Clinical Oncology, *JNCI* Journal of the National Cancer Institute

The process of scientific publication is supposed to be self-correcting. Our findings suggest that despite the additional pressure to rapidly disseminate research on COVID-19 treatment, there is not a high rate of errors that would change interpretation of study results or conclusions. However, we are only able to capture errors that are published in errata; other errors may remain. Thus, our results might underestimate the true number of errors in publications related to COVID-19 therapeutics. In addition, article retractions, representing another major category of error (or malfeasance), were beyond the scope of this review. As reported by Peterson et al., COVID-19 articles have disproportionately retracted over the time span of the pandemic [34].

The COVID-19 pandemic was a sudden, disruptive force to the scientific publication process. We hypothesized that the unprecedented volume of research on a novel disease, the urgent need to disseminate findings, and necessity for accurate and high-quality data would have stress-tested the editorial and review processes leading to a noticeable increase in serious errors. Reassuringly, our findings align with error rates reported among studies examining targeted journals which included all study topics. This finding suggests that journals have been able to maintain publication standards for COVID-19 therapeutics during the pandemic. Increased transparency of changes to a journal’s editorial evaluation of COVID-19 research would be helpful in understanding the resources needed and potential for burnout among editors and reviewers. In addition, future research is warranted to evaluate the sustainability of the recent changes to the editorial and review processes and determine whether new approaches to the publication process could translate to non-COVID-19 research.

### Supplementary Information

**Additional file 1. **MEDLINE search term and Embase search term.
