# Supplementary material for: Does haste make waste? Prevalence and types of errors reported after publication of studies of COVID-19 therapeutics
Source: Syst Rev. 2023 Nov 16;12:216. doi: 10.1186/s13643-023-02381-4 (PMC10652527; doi:10.1186/s13643-023-02381-4)
Supplement: Supplementary file 1 — Additional file 1. MEDLINE search term and Embase search term. [file 13643_2023_2381_MOESM1_ESM.docx]

**Appendix 1:**

**MEDLINE search term:**

((2019 novel coronavirus disease or 2019 novel coronavirus infection or 2019 ncov disease or 2019 ncov infection or 2019-ncov disease or 2019-ncov diseases or 2019-ncov infection or 2019-ncov infections or covid 19 or covid 19 pandemic or covid 19 virus disease or covid 19 virus infection or covid-19 or covid-19 pandemic or covid-19 pandemics or covid-19 virus disease or covid-19 virus diseases or covid-19 virus infection or covid-19 virus infections or covid19 or coronavirus disease 19 or coronavirus disease 2019 or coronavirus disease-19 or disease 2019, coronavirus or disease, 2019-ncov or disease, covid-19 virus or infection, 2019-ncov or infection, covid-19 virus or infection, sars-cov-2 or pandemic, covid-19 or sars cov 2 infection or sars coronavirus 2 infection or sars-cov-2 infection or sars-cov-2 infections or severe acute respiratory syndrome coronavirus 2 infection or virus disease, covid-19 or virus infection, covid-19)) AND (((("Published erratum"[Publication Type] OR "corrigendum" OR "erratum" OR "correction")) AND (Therapy/Broad[filter])))

**Embase search term:**

(('coronavirus disease 2019'/exp OR 'coronavirus disease 2019') OR ('severe acute respiratory syndrome coronavirus 2'/exp OR 'severe acute respiratory syndrome coronavirus 2')) AND ('therapy'/exp OR therapy) AND ([erratum]/lim OR 'corrigendum' OR correction:ti OR errata:ti)
